# The Efficacy of Amniotic Membrane Stem Cell (AMSC) Metabolite Product and Vitamin E for Wrinkles, Spots, and Pores in Photoaging

**DOI:** 10.1155/2020/1584541

**Published:** 2020-08-26

**Authors:** Rahmadewi Rahmadewi, Retha Retha, Dyah Ayu Pitasari, Vidyani Adiningtyas Kusumastanto, Agatha Anindita Ayu Ardhaninggar, Irmadita Citrashanty, Maylita Sari, Menul Ayu Umborowati, Cita Rosita Sigit Prakoeswa

**Affiliations:** Dermatology and Venereology Department, Faculty of Medicine, Universitas Airlangga-Dr. Soetomo Teaching Hospital, Surabaya, Indonesia

## Abstract

**Background:**

It is expected that a combination of amniotic membrane stem cell metabolite product (AMSC-MP) and vitamin E after fractional CO_2_ laser as laser-assisted drug delivery (LADD) will provide better effects in photoaging treatment as the combination reaches the target. This promises an option for photoaging therapy in the future.

**Materials and Methods:**

Sixty women with photoaged skins were involved in this experimental study. They were then divided into two groups. The treatment group received a topical combination of AMSC-MP and vitamin E, and the control group received AMSC-MP alone after fractional CO_2_ laser. The treatment was repeated three times.

**Result:**

The Janus assessment results showed a significant difference in pores in the third observation, and the average pore improvements in the treatment group were better than the control group. Wrinkle, UV spot, and polar spot did not show any significant difference.

**Conclusion:**

A combination of the amniotic membrane stem cell metabolite product (AMSC-MP) and vitamin E after fractional CO_2_ laser as LADD only improves pores in photoaged skins.

## 1. Introduction

Photoaging is a difficult biological process, and it is influenced by a combination of intrinsic and extrinsic factors. By far, ultraviolet radiation (UV) is the most common cause of the photodamage process. Damage caused by solar UV radiation, called photodamage, superimposes chronological aging. Photoaging produces wrinkles, lentigines, keratosis, dyspigmentation, telangiectasia, decreased elasticity, texture, and pale color. Appearance of the aging skin, especially in women, can cause mood disorders and decrease self-confidence, affecting the quality of life [1–3].

Indonesia is a tropical country with all-year sun exposure. This means that the Indonesian population is susceptible to photoaging. Most of the world's adult population desires to maintain the appearance of young skin, and this has promoted multibillion industries such as cosmetics, oral or topical cosmetics, prescription drugs, and invasive and noninvasive procedures [4].

Stem cells are important cells to the skin because they are the source for continuous regeneration of the epidermis. Stem cells have the potentials of self-renewal and differentiation. Stem-cell therapy has prevented disease and promoted tissue repair. Human stem cells release various growth factors such as cytokines and several other extracellular matrix (ECM)-regulating agents including type 1 and 3 gene colagenes, elastin, and fibronectin in human dermal fibroblasts (HDFs) [5].

The human placenta, which is routinely discharged postpartum, irrespective of its natural aging process, is still a rich source of stem cells capable of proliferating and in vitro differentiating in many directions. In addition to homing and differentiation in the area of injury, mesenchymal stem cells have a robust paracrine effect, which stimulates the repair process [6]. In the culture medium of amniotic membrane stem cell (AMSC), there are several bioactive elements such as cytokines and growth factors—metabolite products. Growth factors have been proven beneficial for wound healing. This generates an idea to expand its function for skin rejuvenation. The use of growth factors for skin rejuvenation is now widely researched [7–9].

Laser-assisted drug delivery (LADD) involves selective destruction of the layers of the epidermis and dermis to allow penetration and absorption of topical drugs as well as large molecular weight drugs. LADD is fractionally ablative, and it functions by creating a focused zone of damage to selective episodes so that the dermis is more receptive to topical medications.

The efficacy of topical therapy depends on the ability of the therapeutic drug to achieve its targets. Large lipophilic and hydrophilic drugs cannot penetrate the normal, intact skin. Strategies for increasing topical drug administration include chemistry (solvents and surfactants), biochemistry (nanoparticles and inhibitors of lipid synthesis), and physical methods (exfoliation, sonophoresis, and microneedling). It is expected that as the penetration reaches the target, the effects of therapy will last longer. This is more efficient for patients, and it can be a promising photoaging therapy in the future [10].

Vitamin E is popular among the skincare industries due to its antioxidant properties. Vitamin E has been used for more than 50 years in experimental and clinical use in the field of dermatology. Most experimental studies have shown photoprotective, antitumorigenic effects, and the ability to stabilize the skin barrier [11]. Vitamin E can significantly reduce erythema, edema, and skin hypersensitivity related to UVB radiation, but it can also inhibit the appearance of skin cancer induced by UV radiation. Topical vitamin E has emollient, nonirritating properties, permeation ability, and molecules compatible with the skin [12]. Vitamin E usually used in topical products at the concentration from 0.2% to 1.5% [13].

This study aimed to determine the effect of the topical application of amniotic membrane stem cell metabolite products (AMSC-MPs) and vitamin E mixture compared to AMSC-MP without vitamin E with fractional CO_2_ laser as LADD.

## 2. Materials and Methods

### 2.1. Research Design and Subject

This was an experimental study with a controlled clinical trial method. This study compared a combination of topical AMSC-MP and vitamin E (treatment) and topical AMSC-MP without vitamin E (control). Laser fractional CO_2_ was used as LADD for both topical applications. The sample was selected using consecutive sampling. The sample was individuals with photoaged skins who met the inclusion criteria, and they were outpatients of the Dermatology and Venereology Department, Dr. Soetomo General Hospital, Surabaya. Sixty participants were divided into two groups: the treatment and control groups.

### 2.2. Procedure

AMSC-MP was obtained from the Tissue Bank and Regenerative Medicine Department, Dr. Soetomo Teaching Hospital, Surabaya. This study used *α*-tocopherol as the vitamin E. The AMSC-MP was obtained legally pursuant to the standard for the tissue donor and stem cell culture. The combination was gel with a mixture of 9.2 ml AMSC metabolite products, 0.1 ml vitamin E, and 0.8 mg sodium alginate. The mixture was made at the Tissue Bank and Regenerative Medicine Department, Dr. Soetomo Teaching Hospital, Surabaya. The mixture was dosed 2 ml per application measured using a 3 ml sterile syringe.

All the participants had understood the research procedure before the treatment began. The participants were examined using a Janus 3D Facial Analysis System before having treatment to obtain objective baseline data (observation 1). We measured wrinkle, polarized spot, UV spot, and pore category. Thereafter, all participants had facial skincare (priming) with tretinoin 0.025% cream and SPF 30 sunscreen cream for two weeks before treatment.

The combination of AMSC-MP and vitamin E or the AMSC-MP alone was applied after the laser fractional CO_2_ laser by Fraxis® as LADD. The laser parameter was 6.6–10 mJ with 1 stack, 1 pass, and 1 mm distance. Both groups were treated 3 times with an interval of 4 weeks. All participants were instructed to use sunscreen every day and tretinoin 0.025% every day and start from the first week after treatment. The progression of wrinkle, polarized spot, UV spot, and pores was assessed on 4th week after first treatment (observation 2) and 4th week after the third treatment (observation 3).

### 2.3. Statistical Analysis

This study used SPSS software for analyzing the obtained data with a comparative statistical test. The level of significance was *P* < 0.05.

## 3. Results

This study involved a total of 60 individuals with photoaged skins who met the inclusion and exclusion criteria. All participants were willing to take part in the study by signing information about consent, informed consent, and medical approval sheets. The control group results demonstrated significant outcomes in polar spots and pores. Pores appeared significantly improved at the observation 2-3 and 1–3. Meanwhile, the polar spots have consistently improved, starting from the first observation (Table 1).

The results of the treatment group (AMSC-MP + vitamin E) showed significant improvements in polar spots. The polar spots have significant difference appeared since the second observation and the third observation compared to baseline data (Table 2).

Table 3 shows a comparison of wrinkles, UV spots, polar spots, and pores between the treatment and control groups. The pore status of the treatment group appeared significantly improved on the third observation. Also, the average pore improvements were better in the treatment than the control group. However, wrinkles, UV spots, and polar spots did not show a significant difference.

**Figure 1:**
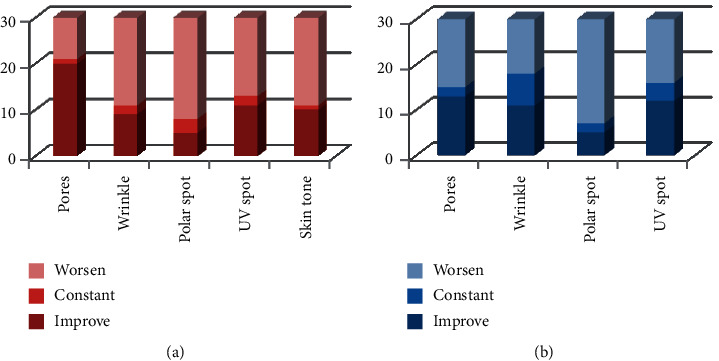
A comparison of the first and second observations between the control (a) and treatment (b) groups.

**Figure 2:**
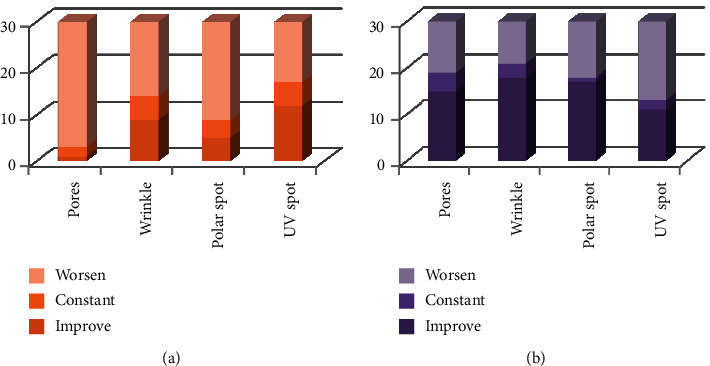
A comparison of the second and third observations between the control (a) and treatment (b) groups.

**Figure 3:**
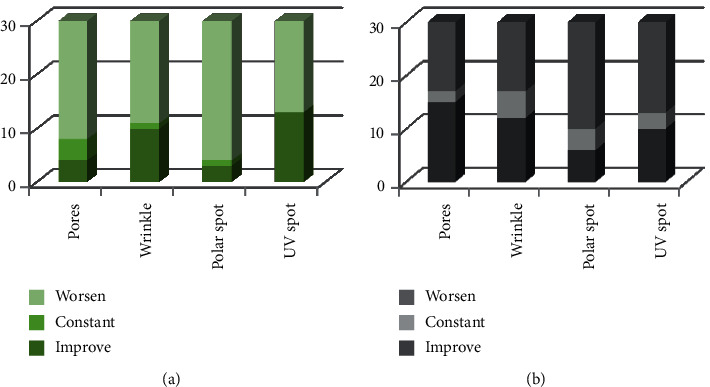
A comparison of the first and third observations between the control (a) and treatment (b) groups.

Figures 1–3 demonstrate a total of value changes for each group in each observation. The changes could be “improve,” “constant,” or “worsen.”

## 4. Discussion

Human placentas can be a source of stem cell metabolites. Compared to adult blood or bone marrow, the human placenta has more hematopoietic stem cells. Metabolite products are produced when stem cells are cultured. Stem cell metabolite products contain several growth factors such as epidermal growth factor (EGF), transforming growth factor-*β* (TGF-*β*), and granulocyte-macrophage colony-stimulating factor (GM-CSF). It also contains cytokines such as interleukin (IL)-4 and IL-10 [14]. Prakoeswa has examined the effectiveness of AMSC metabolite products for photoaging with microneedling compared to placebo and obtained good results [15].

The treatment group (AMSC-MP + vitamin E) demonstrated a significant result in polar spots. The evaluation of polar spots showed a significant difference to the baseline data immediately at the second and third observations. In the third observation, only the pores showed a significant difference. The average pore improvements were better in the treatment than the control group. Wrinkles, UV spots, and polar spots did not show a significant difference.

Our results were in line with a study conducted by Seo et al. on the postmicroneedling fractional radiofrequency and a human stem cell-conditioned medium application. The study results showed an improvement in skin coarseness with histopathology proof of increasing collagen amount with minimal side effects [16]. Lee and colleagues have investigated the comparison between therapeutic products of stem cell metabolites after microneedling and microneedling alone. The study gave significant results on wrinkle repair [7]. Vitamin E can also play a role for wrinkles as antioxidants which are expected to ward off ROS, which can trigger wrinkling formation [17]. Zhou and colleagues' study about the ADSC-BC therapy after fractional CO_2_ laser reported a decreased hyperpigmentation after CO_2_ fractional laser [18]. The research of Lee and his colleagues also reported a significant improvement in the melanin index after therapy using stem cell metabolite products after microneedling [7]. Vitamin E also has an active photoprotection effect that may play a role in repairing black spots due to photoaging [11]. AMSC-MP contains growth factors that can stimulate collagen synthesis, stimulate proliferation and migration of dermal fibroblasts and epidermal keratinocytes, and enhance fibroblast synthesis. This can improve skin elasticity, which then improves the pores. Vitamin E acts as an antioxidant that counteracts ROS by preventing collagen degradation. This can improve the elasticity as well and then further repair the pores.

In conclusion, compared to AMSC-MP alone, the addition of vitamin E has resulted in positive outcomes, the improved appearance of pores on photoaged skins. This study has a potential limitation. It was not certain that the improved pores were not affected by other factors such as maintenance therapy or photoaging triggers.

## Figures and Tables

**Table 1:** Intragroup (the control group) result comparison (AMSC-MP with fractional laser CO_2_ as LADD).

Observation no.	Wrinkle	UV spot	Polar spot	Pores
1-2	0.515	0.332	0.014	0.251
2-3	0.077	0.538	0.003	<0.001
1–3	0.131	0.168	<0.001	<0.001

**Table 2:** Intragroup (the treatment group) result comparison (AMSC-MP + vitamin E with fractional laser CO_2_ as LADD).

Observation no.	Wrinkle	UV spot	Polar spot	Pores
1-2	0.492	0.888	0.001	0.285
2-3	0.246	0.140	0.403	0.362
1–3	0.831	0.050	0.003	0.920

**Table 3:** A comparison between the control and treatment group.

Obs	Wrinkles			UV spots			Polar spots			Pores		
	AMSC-MP	AMSC-MP + vit E	*P*	AMSC-MP	AMSC-MP + vit E	*P*	AMSC-MP	AMSC-MP + vit E	*P*	AMSC-MP	AMSC-MP + vit E	*P*
1	6.73 (2.58)	7.17 (3.75)	0.604	7.77 (3.59)	6.93 (4.43)	0.309	28.77 (6.53)	28.77 (7.96)	1	49.60 (4.92)	49.60 (5.575)	1
2	7.10 (2.98)	7.97 (4.57)	0.547	8.67 (5.12)	7.67 (5.35)	0.350	31.00 (6.22)	32.03 (8.51)	0.593	48.83 (4.95)	50.17 (5.21)	0.314
3	8.47 (4.48)	7.03 (3.63)	0.305	9.07 (5.23)	9.27 (6.37)	0.755	33.90 (6.37)	31.13 (8.74)	0.167	52.40 (5.34)	49.53 (5.14)	0.039

## Data Availability

The data used to support the findings of this study are available from the corresponding author upon request.
